# Assessing the Effectiveness of Antibiotic Irrigation to Reduce Bacterial Load at the Spinal Surgical Site: An In-Vitro Study

**DOI:** 10.7759/cureus.81519

**Published:** 2025-03-31

**Authors:** Rawan Masarwa, Ofir Uri, Abed Athamna, Sarit Freimann, Ali Yassin, Elie Najjar, Rodrigo Muscogliati, Weronika Nocun, Eyal Behrbalk

**Affiliations:** 1 Centre for Spinal Studies and Surgery, Queen's Medical Centre, Nottingham University Hospitals NHS Trust, Nottingham, GBR; 2 School of Medicine, Hull York Medical School, York, GBR; 3 School of Medicine, University of Nottingham, Nottingham, GBR

**Keywords:** intra-operative antibiotic prophylaxis, in-vitro study, post-operative wound infections, spine surgery, surgical site infection, surgical site irrigation

## Abstract

Introduction

Intra-operative surgical site irrigation with antibiotics is believed to reduce the risk of infection in spine surgeries involving instrumentation. However, despite its frequent use, there is limited supporting evidence for this practice. This prospective in-vitro study aims to evaluate the effect of short-term antibiotic exposure on the growth of common pathogens associated with wound infections. Furthermore, this study aims to determine the optimal duration of antibiotic exposure to eradicate common surgical site infection organisms.

Methods

A suspension of one of three micro-organisms: (1) *Staphylococcus aureus*, (2) *Staphylococcus epidermidis*, or (3) *Pseudomonas aeruginosa* was added to 2-ml vials of an enriched medium, containing one of three antibiotics: (a) Vancomycin, (b) Gentamicin, or (c) Cefazolin. The final inoculum of each micro-organism was 10^3^ CFU/ml, representing a contaminated surgical wound in spine surgery. Antibiotics were washed out from the suspension by a centrifugation technique after (i) 5 minutes, or (ii) 8 hours. The recovery of growth of the micro-organisms was monitored by laser light scattering technology.

Results

*P. aeruginosa* inoculated in vials with Gentamicin showed no 24-hour bacterial growth after 5-minute and 8-hour exposure to the antibiotic. Vials of all other bacteria-antibiotic combinations showed bacterial growth curves similar to the control vials after both 5-minute and 8-hour exposures to antibiotics, with no signs of bacterial growth inhibition.

Conclusion

The study demonstrated that Gentamicin effectively inhibited *P. aeruginosa* growth after both short-term (5-minute) and long-term (8-hour) exposures. However, no significant bacterial growth inhibition was observed with other bacteria-antibiotic combinations, regardless of the exposure time. These findings suggest that while Gentamicin may be effective against *P. aeruginosa* in the context of surgical site irrigation, the use of Vancomycin and Cefazolin does not appear to provide the same level of effectiveness for the other tested pathogens. Further studies are needed to evaluate alternative antibiotic strategies for broader infection control in spine surgeries.

## Introduction

Surgical site infections (SSIs) are a serious complication in spine surgery, especially in procedures involving instrumentation, as they are associated with high morbidity and mortality [1]. Moreover, SSI complications following spine surgeries lead to increased healthcare costs, ranging from $12,600 to $38,700, related to extended rehabilitation, prolonged antibiotic therapy, multiple surgical procedures for wound debridement, and implant revision [2, 3].

Despite the wide use of preoperative prophylactic antibiotic treatment and aseptic techniques, SSIs still occur at a rate of 0.6%-12% in patients undergoing instrumented spinal surgery [4-6]. Risk factors for the development of postoperative spinal SSIs include disease severity, number of vertebral levels involved, presence and duration of postoperative inserted drains, prior hospitalization, duration of hospitalization and surgery, and number of transfusions and surgeons [4-8].

Endogenous pathogens, the main source responsible for SSIs, are mostly located on the skin or within the operated organ. According to the European Centre for Disease Prevention and Control (ECDC), *Staphylococcus aureus* is the most common cause of SSIs, with 34.2% of infections being caused by methicillin-resistant *Staphylococcus* aureus (MRSA) strains [9]. This is followed by coagulase-negative staphylococci species, such as *Staphylococcus epidermidis*, causing 13.4% of SSIs [9]. A multicenter analysis identified *S. aureus*, coagulase-negative staphylococci, and *Pseudomonas aeruginosa* as the most common pathogens causing SSIs following spinal instrumentation surgery [10].

The choice of the most optimal antibiotic therapy requires careful consideration of the antibiotic resistance profile, exposure time, dosing, side-effect profile, and patient-related factors such as allergies and comorbidities [11]. Cefazolin, a first-generation cephalosporin, is preferred as it can rapidly reach peak serum concentrations and has fewer side-effects compared to other antibiotics [12]. In cases where there is a high risk of MRSA infection, a combinatorial approach of vancomycin along with a cephalosporin (e.g., cefazolin) or an aminoglycoside (e.g., gentamicin) is recommended according to current clinical practice guidelines for the prevention of SSIs [13]. For patients who are allergic to cephalosporins, vancomycin may be used alone or with an aminoglycoside such as gentamicin [11].

Given the prevalence of gram-positive organisms in SSIs following spine surgery, the application of intrawound vancomycin has been considered as a strategy to reduce infection rates [3]. While this practice has been widely adopted worldwide, recent evidence has raised questions about its efficacy. A recent meta-analysis of randomized controlled trials by Daher M et al. found no significant evidence that the use of vancomycin powder reduces the incidence of SSIs compared to controls [14]. These findings challenge the common assumption that vancomycin is effective in lowering SSI risk in spine surgery, suggesting that further investigation is necessary to ascertain its actual effect.

While some studies suggest that intra-operative surgical site irrigation with antibiotics may reduce the risk of infection in orthopedic and spine surgeries involving instrumentation, the evidence remains variable and inconclusive [3, 15]. Furthermore, although antibiotic irrigation has been widely adopted to prevent SSIs in instrumented spinal surgeries, limited data exist regarding the optimal exposure time to antibiotics. However, short-term antibiotic exposure may be beneficial in reducing risks such as systemic toxicity and the development of resistance associated with prolonged therapy [16].

Despite the widespread use of antibiotic prophylaxis, strong evidence supporting the ideal timing for antibiotic administration is lacking in the literature. Few studies have compared the effectiveness of short-term antibiotic exposure to prolonged therapy. This study aims to evaluate the effect of timed exposure to antibiotics (vancomycin, gentamicin, and cefazolin) on the growth of common pathogens involved in SSIs in spine surgery at Minimal Inhibitory Concentrations (MIC). While antibiotic susceptibility assays are typically conducted at 1 MIC, the study by Topaz M et al. [17] has shown that a higher concentration of 4 MIC could lead to a decrease in exponential bacterial growth. Therefore, our study aims to investigate the impact of higher concentrations of antibiotics, 40 times the MIC, to inhibit the growth of high bacterial loads (10^6^ CFU/ml), as this could better simulate typical intrawound antibiotic irrigation during surgery.

## Materials and methods

Preparation of study materials: microorganisms and antibiotics

A suspension of one of three microorganisms: (1) *S. aureus* (ATCC 25913™), (2) a clinical isolate of *Staphylococcus epidermidis*, or (3) *Pseudomonas aeruginosa* (ATCC 27853™) was added to 2-ml vials of an enriched medium, containing one of three antibiotics: (a) Vancomycin, (b) Gentamicin, or (c) Cefazolin [18]. The antibiotics were diluted in 0.9% w/v sodium chloride solution (normal saline) to the desired 40 MIC for each antibiotic at the standard MIC concentrations for each bacterial strain [17]. Methods of antibiotic dilution and achieving the desired MIC concentration were antibiotic-specific and were performed following guidelines set out by the Clinical and Laboratory Standards Institute, 3rd edition [19].

Bactericidal assay

The antibacterial assay design and protocol were adapted from a recently published study by Topaz M et al. [17]. The bactericidal activities of the antibiotics were determined by monitoring bacterial growth recovery for 24 hours after short exposure, compared to control vials containing microorganisms without antibiotics.

Three to five colonies from overnight growth on blood agar at 35°C were added to normal saline and adjusted to produce a 0.5 McFarland standard suspension of organisms. This suspension was appropriately diluted (1:10) with saline to achieve an inoculum of 10^7^ CFU/ml. A 0.2-ml suspension of an organism was added to 2 ml of enriched medium in vials (final inoculum, 10^6^ CFU/ml) with a 0.2-ml stock solution of each antibiotic at concentrations 40 times the MIC. In some cases, the suspension was diluted 1:10,000 with saline to achieve an inoculum of 10^4^ CFU/ml. A 0.2-ml suspension of an organism was added to 2 ml of enriched medium in vials to a final inoculum of 10^3^ CFU/ml, representing a contaminated surgical wound in spine surgery.

The vials were then placed in the incubated ion chamber at 37°C with constant stirring. After 5 minutes or 8 hours (representing the time of tissue exposure to antibiotics after intra-operative surgical site irrigation or applying antibiotic powder to the surgical field before wound closure, respectively), the vials were removed, and the suspensions were transferred to sterile tubes. The tubes were centrifuged for 10 min at 3500 rpm, and then the supernatant was discarded, and the pellet was suspended in saline, vortexed, and re-centrifuged again (washing 3 times). The washing procedure aimed to reduce antibiotic carryover and to control antibiotic exposure time. The bacterial pellet from the last centrifugation was suspended in 0.5 ml of saline and introduced to fresh vials and re-introduced to the chamber to detect the recovery of bacteria. Growth control vials for each organism were prepared without antibiotics and run in parallel to the antibiotic test vials. The recovery of growth of the microorganisms was monitored for 24 hours by laser light scattering technology using the Uro-Quattro HB&L automated system (AliFax S.r.l., Italy) and compared to control vials containing microorganisms without antibiotics. The various time points that were monitored included 0, 5, 10, 15, 30, and 60 minutes, as well as 2, 4, 8, 18, and 24 hours.

## Results

Incubation of *P. aeruginosa* with Gentamicin inhibited the bacterial growth for 24 hours after 5-minute and 8-hour exposures to the antibiotic. Vials of all other bacteria-antibiotic combinations did not demonstrate any growth inhibition, and growth curves were similar to those of the control cultures, in which the bacterial cultures were not exposed to any antibiotic.

**Figure 1:**
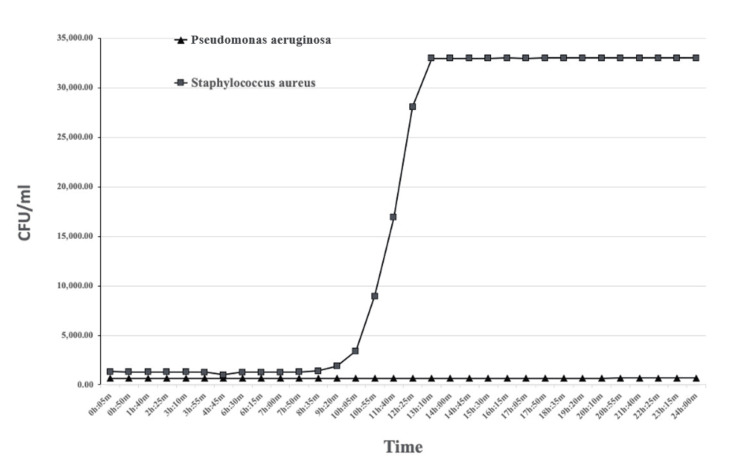
Growth curves of Pseudomonas aeruginosa and Staphylococcus aureus 24 hours after 5-minute exposure to gentamicin.

Data for growth are presented in Table 1. Examples of growth curves for 24 hours after exposure of *P. aeruginosa* and *S. aureus* for 5 minutes to Gentamicin are presented in Figure 1.

**Table 1:** Growth (presence '+' or absence '-') of pathogens (Pseudomonas aeruginosa, Staphylococcus aureus, Staphylococcus epidermidis) over 24 hours after 5 minutes or 8 hours of exposure to various antibiotics (gentamicin, vancomycin, and cefazolin).

	Exposure time	5 minutes				8 hours		
		Gentamicin	Vancomycin	Cefazolin		Gentamicin	Vancomycin	Cefazolin
Pseudomonas aeruginosa	Time from exposure							
	0	+	+	+		+	+	+
	5 minutes	-	+	+		-	+	+
	10 minutes	-	+	+		-	+	+
	15 minutes	-	+	+		-	+	+
	30 minutes	-	+	+		-	+	+
	60 minutes	-	+	+		-	+	+
	2 hours	-	+	+		-	+	+
	4 hours	-	+	+		-	+	+
	8 hours	-	+	+		-	+	+
	18 hours	-	+	+		-	+	+
	24 hours	-	+	+		-	+	+
	Staphylococcus aureus	0	+	+	+		+	+	+
5 minutes		+	+	+		+	+	+
10 minutes		+	+	+		+	+	+
15 minutes		+	+	+		+	+	+
30 minutes		+	+	+		+	+	+
60 minutes		+	+	+		+	+	+
2 hours		+	+	+		+	+	+
4 hours		+	+	+		+	+	+
8 hours		+	+	+		+	+	+
18 hours		+	+	+		+	+	+
24 hours		+	+	+		+	+	+
Staphylococcus epidermidis		0	+	+	+		+	+	+
	5 minutes	+	+	+		+	+	+
	10 minutes	+	+	+		+	+	+
	15 minutes	+	+	+		+	+	+
	30 minutes	+	+	+		+	+	+
	60 minutes	+	+	+		+	+	+
	2 hours	+	+	+		+	+	+
	4 hours	+	+	+		+	+	+
	8 hours	+	+	+		+	+	+
	18 hous	+	+	+		+	+	+
	24 hours	+	+	+		+	+	+

## Discussion

The current study demonstrated that Pseudomonas aeruginosa, a common gram-negative pathogen responsible for spinal SSIs, exhibited no growth following both 5-minute and 8-hour exposures to Gentamicin. This finding corroborates Gentamicin’s efficacy even with short-term exposure, supporting the notion that a brief application of antibiotics during surgery can prevent bacterial growth. Previous studies, such as the meta-analysis by Chang WK et al., have similarly found Gentamicin to be effective in reducing the incidence of SSIs when applied locally to surgical wounds [20].

In contrast, other bacteria-antibiotic combinations in the study, including *S. aureus* and *S. epidermidis* with Vancomycin or Cefazolin, showed no significant growth inhibition after 5-minute and 8-hour exposures. This lack of inhibition suggests that these antibiotics may require longer exposure times or higher concentrations to achieve similar antibacterial effects in clinical settings. These findings are in line with the variable evidence reported in previous literature regarding the efficacy of vancomycin powder in reducing SSIs in spine surgeries [14].

The results suggest that short-term irrigation with Gentamicin could be a valuable addition to current SSI prevention strategies, as this study has shown that in-vitro use of Gentamicin has led to an inhibition in bacterial growth, particularly for gram-negative organisms like *P. aeruginosa*. Given the risks associated with prolonged antibiotic exposure, such as systemic toxicity and the development of resistant bacterial strains, short-term application could provide a safer and equally effective alternative. However, for gram-positive bacteria such as *S. aureus* and *S. epidermidis*, additional strategies, such as prolonged exposure or combination therapies, may be necessary to achieve optimal infection control.

One limitation of this study is that it focused on in vitro models, which may not fully capture the complex in vivo dynamics of infection prevention in surgical wounds. Additionally, the stringent washing steps to prevent antibiotic carryover could have reduced the observed efficacy of Vancomycin and Cefazolin. Future studies should explore the effects of different exposure times, concentrations, and combined therapies in vivo to better understand the potential of short-term antibiotic applications in preventing SSIs. Moreover, a combination of local and systemic antibiotics could be investigated to enhance the effectiveness against a broader range of pathogens.

## Conclusions

This study provides evidence that short-term exposure to Gentamicin can effectively inhibit *P. aeruginosa* growth, supporting its use in SSI prevention during spine surgeries. However, further research is necessary to optimize antibiotic use against gram-positive organisms and to confirm these findings in a clinical setting.
